# Effects of physical exercise during pregnancy on delivery outcomes: Systematic review and meta-analysis of randomized controlled trials

**DOI:** 10.1371/journal.pone.0326868

**Published:** 2025-07-23

**Authors:** Betelhem Abebe Andargie, Abdrehman Legas, Abebe W/Sellassie, Habtamu Abuhay, Dessie Abebaw Angaw

**Affiliations:** Department of Epidemiology and biostatistics, Institute of public Health, College of Medicine and Health Sciences, University of Gondar, Gondar, Ethiopia; Kasr Alainy Medical School, Cairo University, EGYPT

## Abstract

**Background:**

Pregnant women should engage in a range of physical activities as it is not only safe but also has many maternal benefits. This systematic review and Meta -analysis aimed to assess the pooled effect of physical exercise during pregnancy on delivery outcomes.

**Method:**

We conducted a systematic literature search on PubMed, Cochrane Library, Science Direct, Embase, Scopus, HINARI, PsycINFO, Google Scholar, and backward and forward citations for studies published since 2010. Only randomized controlled trials (RCTs) published in English, assessing the effect of exercise on the mode of delivery as a primary or secondary outcome, were included. Quality assessment was performed using Cochrane guidelines, and the certainty of evidence was evaluated using the GRADE approach. A random-effects model was used for meta-analysis, and results were reported as risk ratio (RR) and weighted mean difference (WMD) with 95% confidence intervals (CIs). Heterogeneity was assessed using I² statistics, and potential publication bias was examined. The primary outcome was the mode of delivery, while secondary outcomes included duration of labor, Apgar score, and birth weight.

**Results:**

A total of 16 RCTs involving 3,387 women (1,704 exercise and 1,683 control)were included. Compared to the control group, pregnant women with exercise interventions had a higher rate of normal vaginal delivery (RR = 1.14, 95% CI: 1.08–1.21, I² = 25.6%, moderate certainty of evidence) and a significantly lower rate of cesarean delivery (RR = 0.66, 95% CI: 0.55–0.80, I² = 22.47%, high certainty of evidence). However, no statistically significant association was found between exercise and instrumental delivery (RR = 0.84, 95% CI: 0.70–1.02, moderate certainty of evidence). The mean duration of the first stage of labor was significantly shorter in the exercise group (WMD = −61.30 minutes, 95% CI: −80.63 to −41.97, moderate certainty of evidence). However, no significant differences were observed for second stage of labor birth weight, Apgar scores at one and five minutes (moderate certainty of evidence).

**Conclusion:**

This systematic review and meta-analysis consolidate existing evidence, demonstrating that physical exercise is associated with an increased likelihood of normal vaginal delivery, a reduced rate of cesarean delivery, and a shorter first stage of labor. These findings reinforce the importance of encouraging pregnant women without contraindications to engage in appropriate physical activity during pregnancy, contributing to informing clinical practice.

**Trial registration:**

This study was registered on PROSPERO number CRD42022361132.

## Background

According to the World Health Organization (WHO), physical exercise is any skeletal muscle-driven motion that involves the use of energy [1]. Since nearly all of the body’s control systems undergo distinct modifications during pregnancy to ensure both maternal and fetal equilibrium [2], Pregnant women should engage in a range of physical activities as it is not only safe but also has many maternal benefits [3].

The American Congress of Obstetricians and Gynecologists (ACOG)recommends that all pregnant women engage in 30 minutes of moderate-intensity exercise on most, if not all days of the week, barring obstetric and medical contraindications. Women who were active prior to becoming pregnant may continue to do so, although their frequency and intensity may decrease as the pregnancy progresses [4].The WHO, along with global recommendations for physical activity during pregnancy reviewed in 2020, suggests that pregnant women should limit sedentary time and engage in moderate-intensity aerobic physical activity throughout pregnancy, as it has no known adverse effects [1,5].Contrary to this, a review of physical activity patterns in pregnant women showed that up to 60% of women are physically inactive during this time [6] and only 3 out of every 10 pregnant women meet the physical activity recommendation [7].

The positive effects of physical activity during pregnancy for the mother and the offspring include reduced risk of excessive gestational weight gain and conditions such as gestational diabetes, macrosomia in new born, preeclampsia, and preterm birth. There is also evidence that regular exercise during pregnancy can have additional benefits for labor and delivery. Physically active women tend to have shorter duration of first and second stage labor and normal delivery [8]. By altering metabolic and hormonal changes, uterine contractility, endurance, and strength, exercise during pregnancy influences the mode of delivery. The pelvic floor muscles can be 90% stronger with the right workouts throughout pregnancy, which is crucial for a normal birth [9].

Given its shorter hospitalization, reduced medical intervention and reduced incidence of infection and hemorrhage following birth, compared to cesarean section (C-section), normal delivery is the ideal way to end the pregnancy for the mother and the newborn, both physically and psychologically [10]. Only when necessary should any other than a regular delivery be used during childbirth. These techniques may include caesarean sections and instrumental births with vacuum or forceps [11,12]. Per the recommendations of the WHO [13], only 10–15% of births necessitate caesarean procedures. Nonetheless, the most recent data globally, show that 21.1% of women worldwide underwent caesarean sections. According to projections, 28.5% of women worldwide will give birth through C-section in 2030 [14]. Approximately 10–20% of deliveries around the world require somewhat of a support or interference at delivery, and 6–12% of these interventions are made by IVDs [15].

Considering the procedure’s potential risks to the mother and fetus, this percentage is alarming from the standpoint of public health. In a typical pregnancy, C-section has an eight-fold greater mortality rate and an 8–12 times higher morbidity rate than natural birth [16].Reduced activity during pregnancy is one of the factors contributing to an increase in C-section or IVD rates worldwide [10]. Numerous studies have assessed how exercise affects the method of delivery. Some studies link physical activity to a lower incidence of cesarean deliveries and instrumental deliveries, [17], others revealed no connection between pregnancy exercise and delivery mode [18]. Although there have been significant breakthroughs in scientific understanding and the creation of guidelines to encourage physical activity in pregnancy, the majority of pregnant women do not follow the current physical activity recommendations, and many continue to be sedentary both during and after pregnancy [19].

Despite increasing evidence on the benefits of physical activity during pregnancy, its exact impact on delivery outcomes, particularly the mode of delivery, remains unclear. Research findings have been inconsistent, and there is still no strong consensus on whether exercise significantly influences delivery type. Meanwhile, the global rise in C-section rates underscores the need for cost-effective, non-invasive strategies to promote normal delivery. The lack of a clear synthesis of existing data creates uncertainty in clinical and policy recommendations. Therefore, this systematic review and meta-analysis aim to consolidate the most recent and reliable evidence from randomized controlled trials to determine the overall effect of prenatal exercise on delivery outcomes.

## Methods and materials

A systematic review and meta-analysis was performed. To avoid duplication of ideas the protocol for this systematic review and meta-analysis was registered on International Prospective Register of Systematic Reviews (**PROSPERO CRD42022361132**) and report was designed in accordance with Preferred Reporting Items for Systematic Reviews and Meta-Analysis (PRISMA) 2020 statement [20].

### Eligibility criteria

Eligible studies were published studies that evaluated effect of physical exercise on mode of delivery as primary or secondary outcome and met the following criteria:-Population group: Healthy pregnant women with singleton pregnancy and aged >=18,with normal weight,Intervention: Exercise (any physical activity during pregnancy consisting of planned, structured, and repetitive bodily movements targeted to improve mode of delivery or more components [aerobic,aquatic, yoga, Pilates) according to ACOG, Control group: pregnant women with standard antenatal care and no exercise, Outcome: a randomized controlled trial should Evaluate the outcome of mode of delivery,Study design: published randomized controlled trials (RCTs), Time: studies published since the year 2010 up to date.Setting: no restriction was done according settings, including studies done worldwide. Restriction was done in language, thus this systematic review included those RCTs published in English language only.

We excluded Randomized controlled trial without full text, studies written in languages other than English and pregnant mothers with other comorbid diseases, complicated pregnancy and Studies with multicomponent interventions including exercise.

### Search strategy and selection process

We used many search strategies to broaden the range of research beyond prior reviews and prevent biases generated by limited searches. A search was conducted on PubMed, Cochrane Library, Science direct, Embase, Scopus, HINARI and PsycINFO and on Google scholar search engine. The search strategy included key words identified from the PICO (Population, intervention, comparator and outcome), and mix of Mesh terms, and entry terms identified through Mesh browser and brainstorming (S2 File). The keywords were connected with Boolean operator; Phrases were conjoined by quotation to be considered as a single word, while truncations were used to involve all alternatives of a specific word. The following search terms were used: exercise, pregnancy, mode of delivery. Citations identified from the literature searches and reference list checking was exported to EndNote-7 citation manager software. Two reviewers (AL and BAA) independently screened the title and abstract.In case of discrepancies, consensus was reached on inclusion or exclusion of the study by discussion and if necessary, the third reviewer (HA) was consulted.

### Data extraction process

A data extraction excel sheet was developed; pilot tested on four randomly selected included articles and then refined. After finalizing two reviewers (AL and BAA) performed the initial data extraction for all included articles and the rest two reviewers (HA or AW) verified the data extraction. We reached consensus for the following data: General study information (first author, year of publication, country, continent, Design type, sample size with attrition rate), participant characteristics (Age, BMI, educational status,occupation and obstetric characteristics,), detail information on the intervention (type, session length,duration, frequency.intensity of physical exercise and GA at start, history of exercise before pregnancy) Study outcomes (primary outcome: mode of delivery and secondary outcomes: duration of labour, Apgar score & birth weight) and Quality assessment.

### Data items and outcome measurement

Mode of delivery was the primary outcome defined as occurrence of birth in either Normal Vaginal Delivery, assisted vaginal or cesarean [21].While Birth weight,Apgar score and duration of Labor were secondary outcomes. Since all included studies use similar measurement weighted mean differences (WMD) effect sizes (mean difference)was calculated for the continuous outcomes and risk ratios with 95% confidence intervals (CIs) for outcomes of individual RCTs whenever possible.

### Study risk of bias assessment

We assessed risk of bias in the included studies using the revised Cochrane ‘Risk of bias’ tool for randomized trials (RoB2.0) [22]. The tool addressee’s seven specific domains: (1) the sequence generation process; (2) concealment of randomization (3) blinding of participant & personnel (4) blinding of outcome assessor (5) incomplete outcome data (6) selection of the reported result and (7) other biases. RevMan software version 5.4.1 was used to explore assessment of risk of.

Two review authors (AL and BAA) independently assessed the quality using the tool to each included study, and recorded supporting information and justifications for judgments of risk of bias for each domain (low; high or unclear). Any discrepancies in judgments of risk of bias or justifications for judgments were resolved by discussion to reach consensus between the two review authors, with a third review author (AW) acting as an arbiter if necessary.

### Data synthesis and analysis

We used Microsoft Excel 2010 to extract data, which was then exported to STATA version 17.0 for a full meta-analysis. Where trials were deemed sufficiently homogenous in design, participant characteristics, and interventions; fixed‐effect Meta‐analysis using Mantel‐Haenszel was performed for categorical outcomes and inverse‐variance methodology for continuous outcomes. The meta‐analysis was repeated with estimation of restricted maximum likelihood random‐effects model through examining forest plots when evidence of heterogeneity was found.

We assessed clinical heterogeneity by reviewing the differences across trials in design, characteristics of recruited participants, and interventions. Heterogeneity was statistically assessed using a Chi^2^ test for heterogeneity (with a conservative judgment of P value less than 0.05 suggesting heterogeneity), and the I^2^ statistic. We interpreted the I^2^ statistic as follows [23].0% to 25% might not be important; 25% to 50% may represent moderate heterogeneity; 50% to 75% may represent substantial heterogeneity;75% to 100%: considerable heterogeneity. Leave one out Sensitivity analysis was carried out to analyze the influence of each study on the overall results, each study would be deleted from the model once and the pooled analyses were conducted without this study in the model.

Finally,as the presence of a publication bias will result in an overestimation of meta-analysis results [24], we visually inspected funnel plots for each meta-analysis when the required statistical conditions were met (≥ 10 studies and no significant heterogeneity) [25] and then statistical Method (Harbord regression test & Duval and Tweedie’s The Trim and Fill) were used. If a publication bias exists, the p value is below 0.05 in the harbord’s regression intercept test [26].

### Summary of findings & certainty assessment

The level of confidence of the evidence was separately determined by two persons (AL and BAA). The GRADE approach [27], which includes five GRADE considerations (study limitations(risk of bias), consistency of effect, imprecision, indirectness, and publication bias) to assess the certainty of the body of evidence as it related to the studies that provided data to the meta-analyses for the pre specified outcomes, was used to determine the degree of certainty of the evidence. We gave evidence a high, moderate, low, or very low certainty rating. We used footnotes to support every choice to downgrade or upgrade the confidence of findings.

## Result

### Study selection

**Fig 1:**
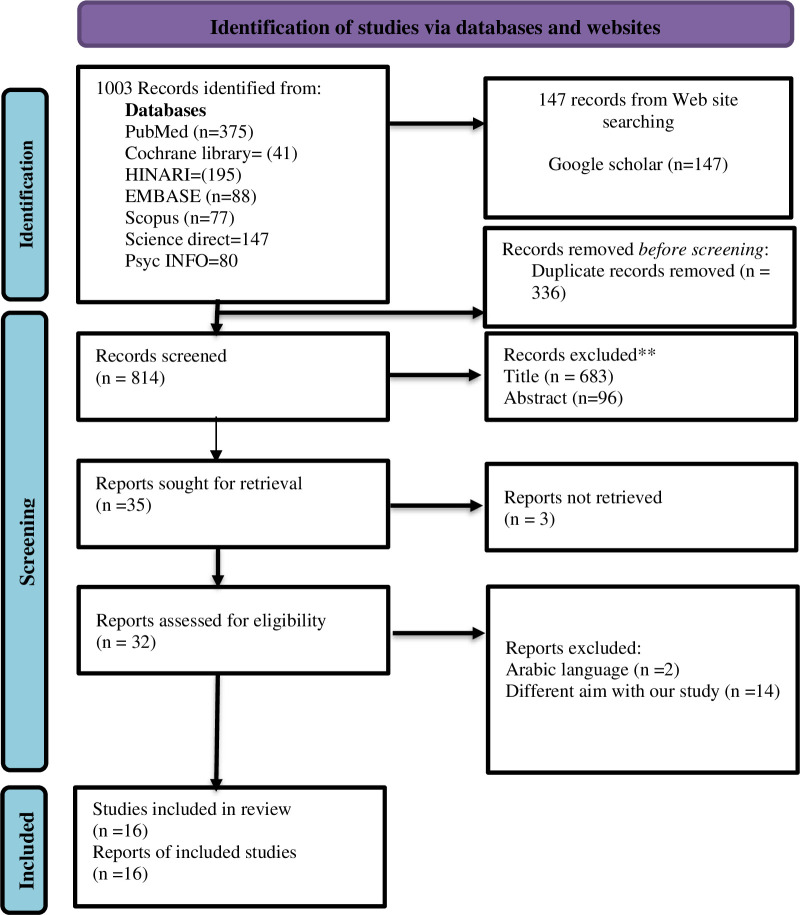
Preferred Reporting Items for Systematic Reviews and Meta-analyses (PRISMA)2020 Flow diagram of search results, study screening, and study inclusion.

Ten studies were included in the previous meta-analysis [8].The studies to be included in this review were identified during the period of 21 August 2023–26 September 2023. The search strategy resulted in the identification of 1,150 articles in the seven ahead mentioned databases and one search engine (Fig 1). After discarding duplicates, 35 articles were considered for the analysis of titles and abstracts [28,29],of these, 3 publications were not obtainable [29,30–32], other 2 where published in language other than English [33,34], and hence excluded. 14 studies were excluded mainly due to having different aim with our study. In the end, sixteen studies met the pre-established eligibility criteria and were therefore included in this review [28,35–49].

### Study characteristics

The sixteen studies selected to be included [40] in this review were randomized controlled trials published in English after the 2010, which assessed effect of exercise on mode of delivery in pregnant women above 18 years of age as a primary or secondary outcome. Among these studies, ten were conducted in Spain [28,35–39,41,45,46,49], three in Iran [42,44,47], one in the United States, one in Norway [42] and one from India [40].

Studies included in this systematic review and meta- analysis had minimum sample size was 60 [43] and maximum sample size was 840 [36], with six studies enrolling 100 or fewer participants [28,39,44–46,48]. While seven studies reported no lost to follow up [28,38,39,43,44,46,49], the rest nine studies reported loss to follow-up that ranged from 2% to 28% [36,37,40–42,47,45,50,51].

Nine of the studies included in this systematic review reported mode of delivery as their primary outcome [35,38,40,42,43,45–48]. While the rest seven studies were interested in other outcomes, such as duration of labor or labor pain, and mentioned mode of delivery for this review only as secondary outcomes [28,36,37,39,41,44,50].

Interventions varied with regard to exercise type along with the frequency and intensity at which they were performed (Table 1). Among these sixteen studies, nine studies used aerobic training [28,35–39,45,46,48], while four studies used yoga [40,43,44,49]. Two studies reported aquatic exercise [41,42] and the last one studies with Pilates exercise [47]. All studies included in this systematic review described characteristics of their control groups which were consisted of pregnant women with the standard antenatal care with no supervised exercise.

**Table 1:** Intervention of included studies (n = 16).

Intervention							
Studies	Type	Duration [weeks]	Frequency/ week	Session minute/week	Intensity	Trimester	Control group
Barakat et al. 2011	Aerobics	28	3	120	Moderate	1	Standard antenatal care
Barakat et al.[A] 2012	Aerobic	31	3	127	Moderate	1	Standard antenatal care
Barakat et al.[B] 2012	Aerobic	28	3	120	Moderate	1	Standard antenatal care
Barakat et al. 2013	Aerobic	28	3	171	Moderate	1	Standard antenatal care
Barakat et al. 2016	Aerobic	30	3	157	Moderate	1	Standard antenatal care
Barakat et al. 2018	Aerobic	28	3	172	Moderate	1	Standard antenatal care
PRICE et al. 2012	Aerobic	23	4	210	Moderate	2	Standard antenatal care
Silveira et al. 2012	Aerobic	28	2	80	Moderate	1	Standard antenatal care
Perales et al. 2014	Aerobic	28	3	172	Moderate	1	Standard antenatal care
Chethana et al. 2018	Yoga	10	3	90	Moderate	3	Standard antenatal care
Jahdi et al.2016	Yoga	12	3	180	Moderate	3	Standard antenatal care
Mohyadin et al. 2020	Yoga	12	3	180	Moderate	3	Standard antenatal care
Yekefallah et al.2021	Yoga	12	2	150	Moderate	3	Standard antenatal care
Ghandali et al. 2020	pilates	12	2	90	Moderate	3	Standard antenatal care
Carrascosa et al. 2021	Aquatic	21	3	135	Moderate	2	Standard antenatal care
Haakstad et al. 2020	Aquatic	26	2	120	Moderate	2	Standard antenatal care

The studies in these systematic review employed exercise with moderate intensity. Exercise sessions were supervised by a physiotherapist or a fitness specialist. The intervention started during the first trimester in eight studies [28,35–39,45,46] three at second trimester [41,42,48] and the rest five at the third trimester [40,42–44,47], the intervention lasted from 10 weeks to 31 weeks across the studies. Twelve studies planned the intervention for 3 times per week weeks [28,35–41,44,45,48], while four studied implemented for 2 times/week [42,45,47,49]and only one study conducted the intervention for 4 times/week [50]. The included studies had exercise session length ranging from 90–210minutes/week. While only four studies included pregnant women with previous history of physical activity [35,36,38], the rest twelve studies included pregnant women with no previous history of exercise [28,37,39–45,47–49]

### Characteristics of participants

All studies included in this systematic review, had normal mean BMI (BMI, < 25 kg/ m2). The mean age of participants in the studies ranged from 23–32 years. Five studies included nulliparous women [40–42,44,49]; three studies [43,46,47] only included multiparous women while the rest involved both nulliparous and multiparous pregnant women.

### Outcomes of included studies

Regarding to the outcomes all of the studies reported normal delivery among which one study (94%) do not’ report cesarean delivery [35] and five studies do not’ report about instrumental delivery [30%] [43,46–49]. Of the studies in this review twelve of the trials reported Apgar score [70%] [28,35–41,43–45,47,49] and nine trials had reported the outcome mean birth weight of the new born [28,35–39,45,48,49]

with mean gestational age at delivery 272–279 days, while only of the six randomized controlled trials reported mean duration of first stage of labor [35,42–45,47,50] and only Seven studies included second stage of labor [35,42–45,47,50].

### Risk of bias

**Fig 2:**
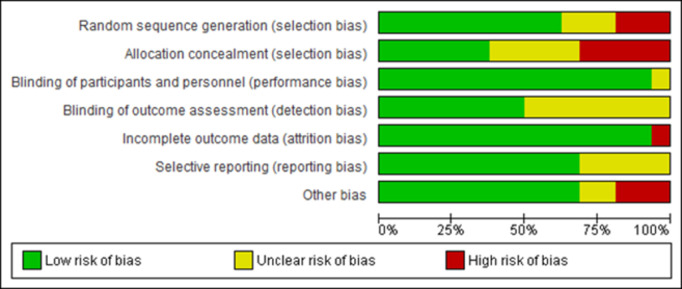
Risk of bias graph: review authors’ judgments about each risk of bias item presented as percentages across all included studies (n = 16).

**Fig 3:**
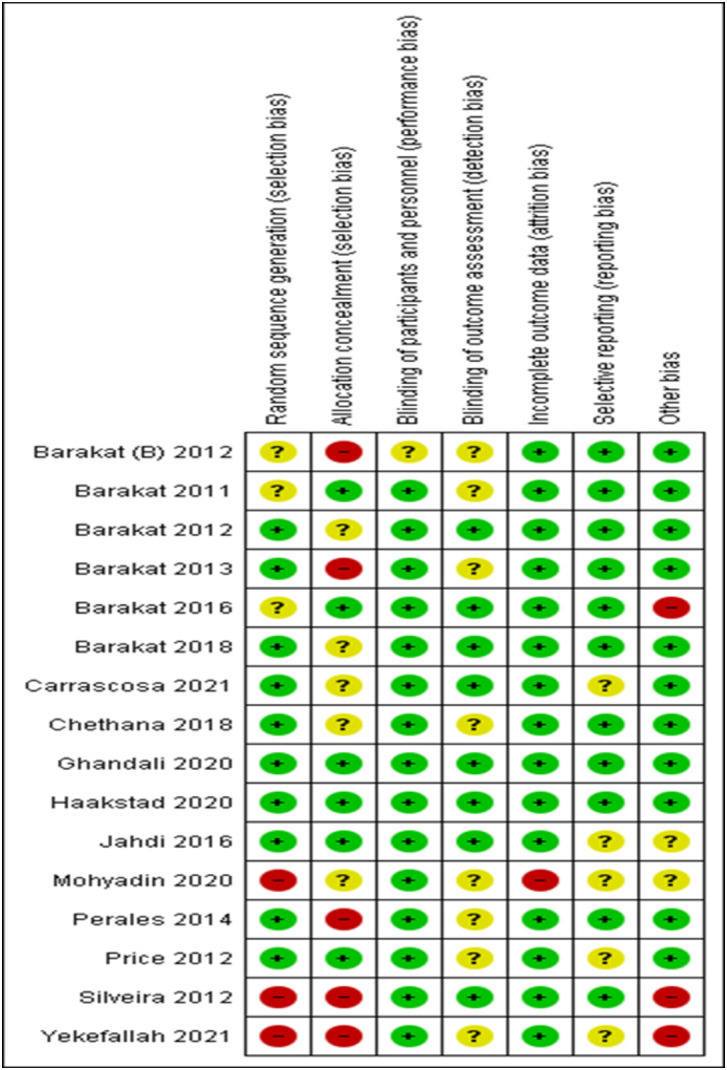
Risk of bias summary: review authors’ judgments about each risk of bias item for each included study [n = 16).

The Review Manager (RevMan) version 5.4.1 software produced a risk of bias graph and summary. Totally three studies of the included trial were of good quality, while two of the studies in this systematic review were of poor quality. Of the sixteen studies included in the review, only three studies were at high risk of bias for sequence generation as it was based on the even/odd days in one study. For the rest of studies methods adopted for sequence generation remained unclear. Allocation was properly concealed in six studies only. Blinding of outcome assessors was low risk of bias in eight studies. Only one trial had incomplete outcome data and women were lost to follow up in both the intervention and the control groups (Figs 2 and 3).

### Random effect meta-analysis

#### Primary outcome.

**Fig 4:**
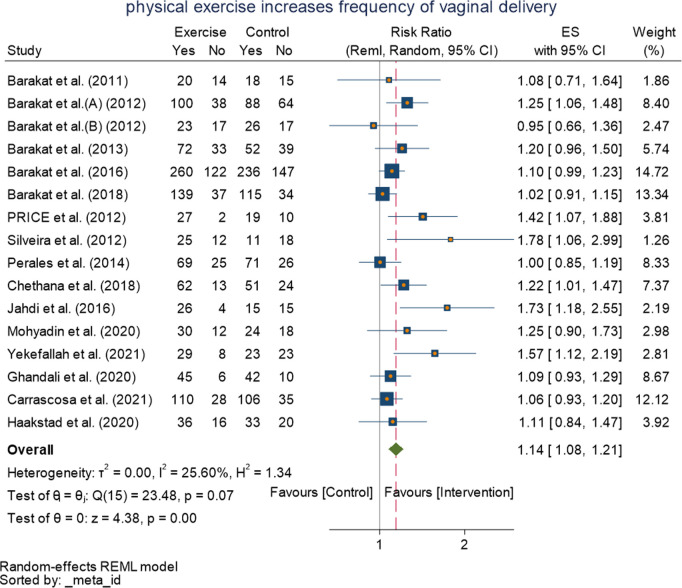
Forest plot of Random effect meta-analysis: Effect of physical exercise during pregnancy on Normal vaginal delivery.

As shown in (Fig 4) all sixteen included RCTs (n = 3387) provided sufficient data for inclusion in the meta-analysis of intervention effect of exercise on normal vaginal delivery, Following meta-analysis of the sixteen included studies, fifteen of these studies reported higher rate of normal delivery in the intervention group compared to the control group, with the exercise group being RRs ranging from 1.02 (95%CI: 0.91–1.15) to 2.33 (95%CI: 1.74–3.14]. Random-effects model with RMLE was conducted,overall significant pooled effect size was found,1.14[1.08,1,21] (I^2^ = 25.60%, p = 0.000 at 95% CI) with no improvement of heterogeneity compared to the fixed effect,this result means the exercise group had a significantly higher rate of normal vaginal delivery compared with the control group.

**Fig 5:**
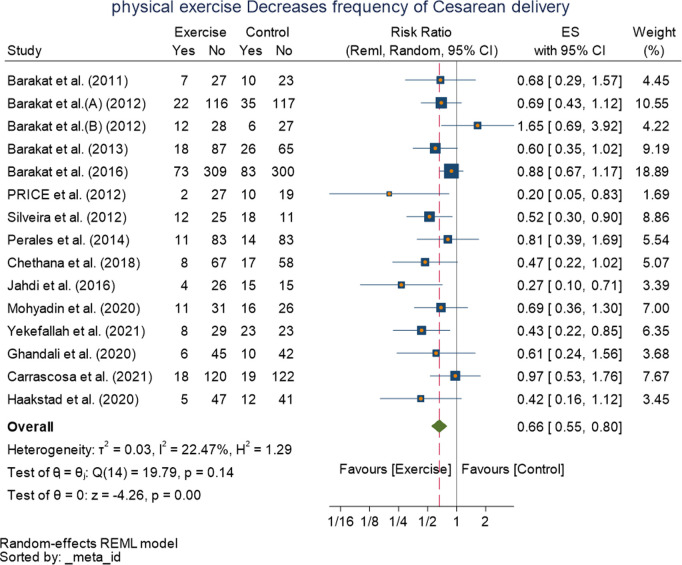
Forest plot of Random effect meta-analysis: Effect of exercise during pregnancy on cesarean delivery.

**Fig 6:**
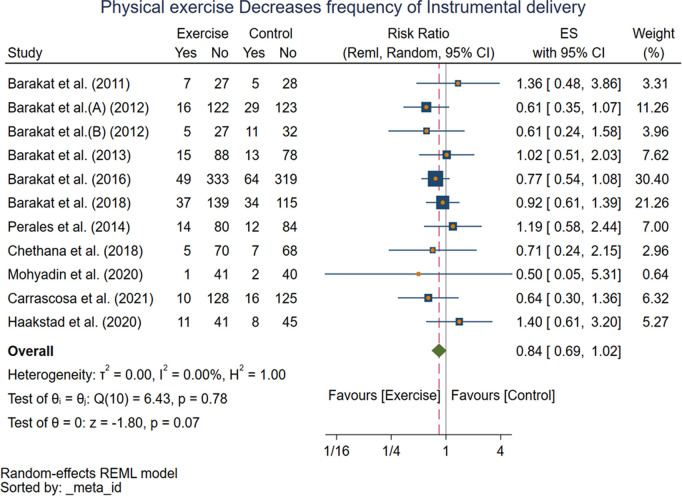
Forest plot of Random effect meta-analysis: Effect of exercise during pregnancy on Instrumental delivery.

Fifteen studies were also available regarding the outcome cesarean delivery.Except for one study 1.65[0.69, 3.92], the studies included had relative risk ranging from 0.20[0.05, 0.83] to 0.97[0.53, 1.76].The random effect model with RMLE method revealed pooled effect size of 0.66[0.55, 0.80] (I^2^ = 22.47%, p = 0.000 at 95% CI), meaning pregnant women in the exercise group had 38% lower risk of cesarean delivery compared with women in the control group. The forest plot shows the pooled effect of exercise on the risk of cesarean delivery (Fig 5). Eleven studies provided available information for instrumental delivery, and it revealed insignificant relationship 0.84[0.69,0.1.02] (I^2^ = 0.00%, p = 0.07 at 95% CI) between exercise and instrumental delivery, suggesting that there is no evidence for an effect of the exercise in improving risk of instrumental delivery (Fig 6).

### Secondary outcomes

#### Apgar score.

**Fig 7:**
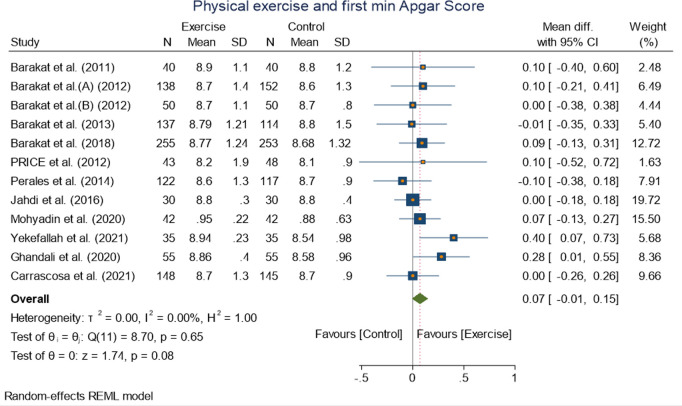
Forest plot of Random effect meta-analysis: Effect of exercise during pregnancy on first minute Apgar score.

**Fig 8:**
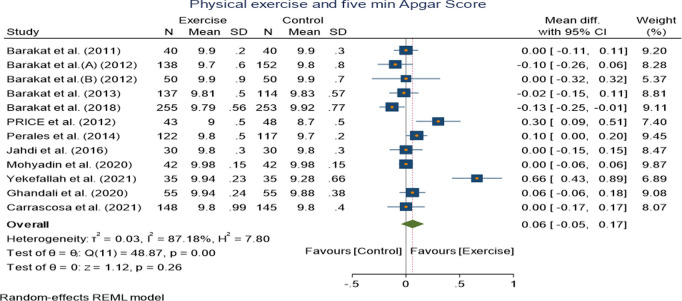
Forest plot of Random effect meta-analysis: Effect of exercise during pregnancy on five minute Apgar score.

Pooled analysis of 12 randomized controlled trials on first minute and five minute Apgar score showed no significant mean difference along the exercise and control group:WMD 0.07[−0.01,0.15] (I^2^ = 0.00%, p = 0.08 at 95% CI) for first minute Apgar score WMD 0.06 [−0.05,0.17] (I^2^ = 87.18%, p = 0.26 at 95% CI) (Figs 7 and 8).

#### Birth weight.

**Fig 9:**
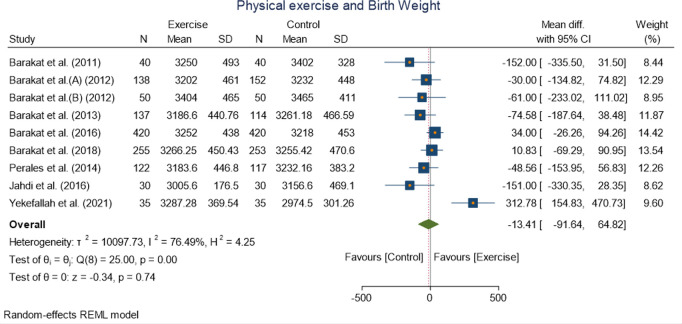
Forest plot of Random effect meta-analysis: Effect of exercise during pregnancy on birth weight.

Nine studies were pooled with 2438 participants in order to see the effect of physical exercise on birth weight for this meta-analysis, revealing a non-significant mean difference with WMD −13.41 [−91.64, 64.82] (p = 0.74 at 95% CI) (Fig 9) was observed. Statistical heterogeneity was high with I(^2^ = 76.49%).

#### Duration of first and second stage of labor.

**Fig 10:**
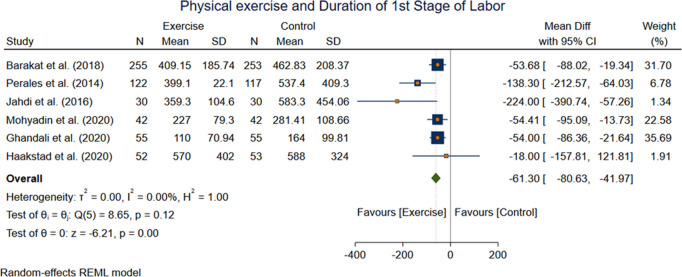
Forest plot Random effect meta-analysis: Effect of exercise during pregnancy on duration of first and second stage labor.

**Fig 11:**
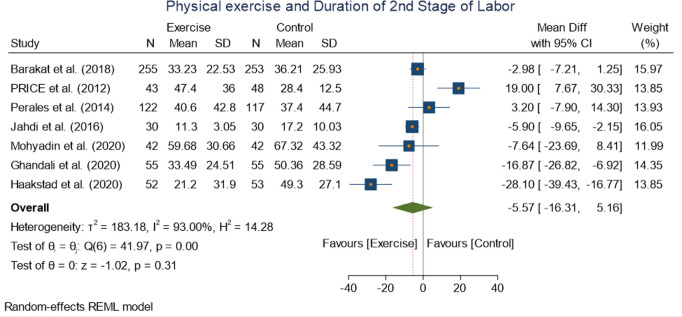
Forest plot Random effect meta-analysis: Effect of exercise during pregnancy on duration of first and second stage labor.

Six studies with 799 participants reported the effect of exercise on the first stage of labor. A significant improvement in duration of first stage of labor among the exercise and control group was observed following an exercise intervention. WMD −61.30[−80.63, −41.97] (p = 0.00 at 95% CI) (Fig 10). There was no evidence of Statistical heterogeneity with I(^2^ = I^2^ = 0.00%).While pooled effect size of Seven studies including 890 participants was insignificant on the second stage of labor between the exercise and control group: WMD −5.57 [−16.31, 5.16] (I^2^ = 93.00%, p = 0.31 at 95% CI) (Fig 11).

### Sensitivity analysis

**Fig 12:**
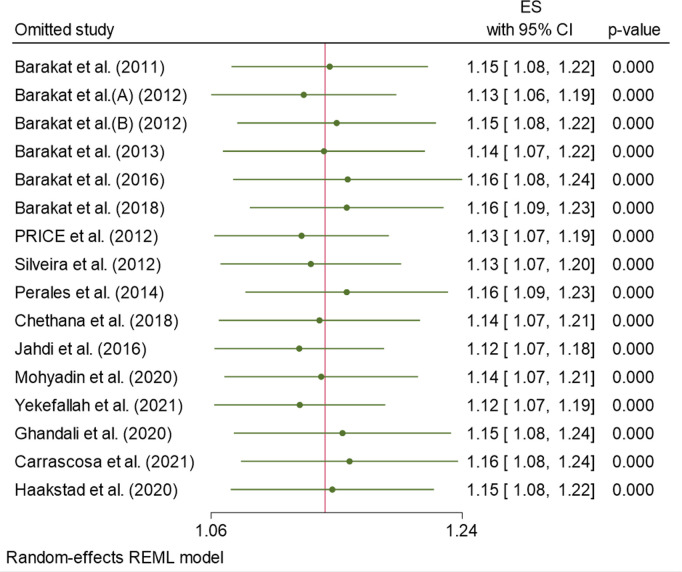
Forest plot of leave-one-out sensitivity analysis of studies on the effect of exercise on Normal of delivery.

The influence of a single study on the overall meta-analysis estimate was employed Based on the point estimate of its omitted analysis lies within the confidence interval of the combined analysis (Fig 12). This suggested that no single study unduly influenced the overall pooled effects of estimate.

### Publication bias assessment

**Fig 13:**
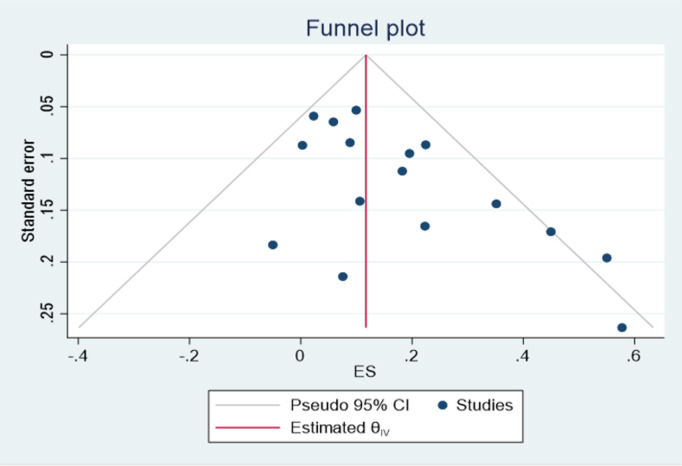
Funnel plot assessing the effect of exercise on normal delivery.

**Fig 14:**
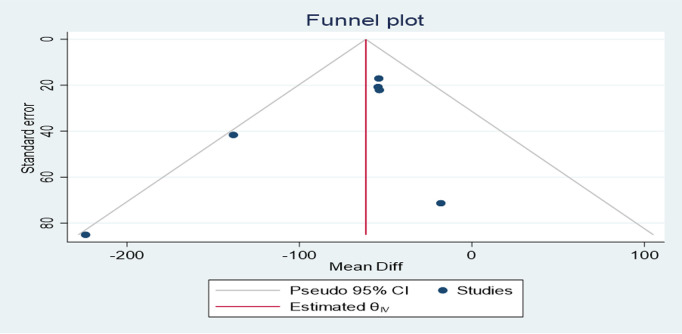
Funnel plot assessing the effect of exercise on cesarean delivery.

**Fig 15:**
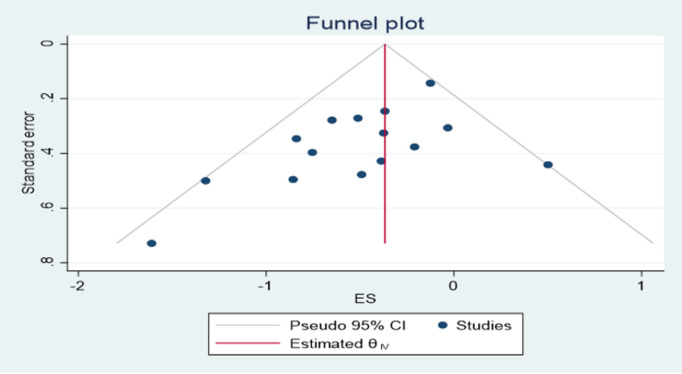
Funnel plot assessing the effect of exercise on duration of first stage of labor.

Asymmetry was detected with a visual inspection of the funnel plot for publication bias for the outcome of normal vaginal delivery (Fig 13), while other outcomes showed no asymmetry in (Figs 14 and 15). Results of the harbord test for outcome of normal vaginal delivery (intercept 1.82; 95% CI,; *P* = 0,0088, t = 3.04) Indicated the presence of small study effect. The relative risk was then adjusted for publication bias according to the Duval and Tweedie trim-and-fill procedure and the pooled effect decreased to (RR = 1.04 (1.02, 1.18)95% CI).While the harbord test were insignificant for the outcome cesarean delivery with (intercept −1.46; 95% CI,; *P* = 0.1010 t = −1.77) showing no publication bias.publication bias was also insignificant for first stage of labor with (intercept −1.52; 95% CI,; *P* = 0.115 t = −1.75).

### Certainty of evidence

We assessed the overall quality of evidence using the GRADE approach (Table 2). According to the assessment of the GRADE approach, the evidence generated has a moderate and high quality of evidence for the statistically significant primary and secondary outcomes.

**Table 2:** Certainty assessment for included outcomes.

Outcome	Study design	Source	Risk of bias	Inconsistency	Directness	Precision	Publication bias	RR[CI]	WMD[CI]	Certainty
Normal delivery	RCT	16	Low[Most information are from studies at low risk of bias]	Not important	Direct	Precise	Present,but handled with trim and fill analysis	1.14[1.08,1.21]		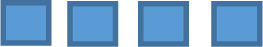 Moderate
Cesarean delivery	RCT	15	Low	Not important	Direct	Precise	No publication bias	0.66[0.55,0.80]		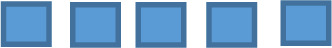 High
Instrumental delivery	RCT	11	Low	Not important	Direct	Imprecise	No publication bias	0.84[0.70,1.02]		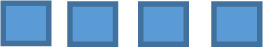 Moderate
Apgar score	RCT	12	Low	Not important	Direct	Imprecise	No publication bias		**0.07[−0.01,0.15]** **0.06 [−0.05,0.17]**	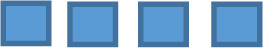 Moderate
Birth weight	RCT	9	Low	Not important	Direct	Imprecise	No publication bias		−13.41[91.64,64.82]	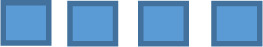 Moderate
Duration of labor	RCT	6	Low	Not important	Direct	precise	No publication bias		−61.30[−80.63, −41.97]	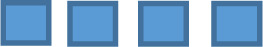 Moderate

**High**: We are very confident that the true effect lies close to that of the estimate of the effect.

**Moderate**: We are moderately confident in the effect estimate: The true effect is likely to be close to the estimate of the effect, but there is a possibility that it is substantially different.

**Low** Our confidence in the effect estimate is limited: The true effect may be substantially different from the estimate of the effect.

**Very low**: We have very little confidence in the effect estimate: The true effect is likely to be substantially different from the estimate of effect.

The quality of evidence was downgraded one level [–1] if the study did not comply with each GRADE criteria.

## Discussion

This systematic review assessed the effects of physical exercise on delivery outcomes. Despite the paucity of research on exercise during pregnancy, it seems that moderate exercise during a low-risk pregnancy improves the mother’s overall health and wellbeing whereas it is not advised to increase physical activity if there is a medical or obstetric issue [52,53]. Conflicting evidence exists about the effects of exercise on maternal and neonatal outcomes.

This meta-analysis of sixteen randomized controlled trial studies including with 3387 pregnant women 1704 on intervention and 1683 on control group examined the impact of supervised and structured prenatal exercise in healthy, normal weight, uncomplicated, singleton pregnant women on mode of delivery, birth weight, Apgar score and duration of labor. As shown in this study exercise during pregnancy significantly improves the likelihood of normal vaginal delivery by 14% and lower incidence of caesarean delivery by 34% caesarean delivery whereas there is no difference with risk of Instrumental delivery. Elsewhere, it has been proposed that exercise may improve maternal and neonatal outcome [54]. The meta-analysis findings also revealed exercise during pregnancy significantly reduces the mean duration of the first stage of labor.. However, there are no appreciable differences between the control and prenatal exercise groups in terms of difference in mean duration of the second stage of labor, Apgar score and birth weight.

Although funnel plots and the harbord test indicated that publication bias might have affected our analyses, our results can be considered robust, since we still obtained significant summary estimates after correction for potentially missing studies using trim and fill for the effect of exercise on normal delivery. Hence, the pooled effect size decreased to 1.04(1.02, 1.18). Regarding heterogeneity, the I2 parameter fell into a low range, according to Higgins, Thompson, and Deeks & Altman suggesting that a large portion of variation in estimates was due to error.

Several studies have examined the effects of physical exercise in pregnant women as their primary or secondary outcome. A review conducted in 2015 assessed the impact of exercise on the type of delivery and showed that prenatal exercise slightly increases the frequency of vaginal birth [8]. Other reviews [54–57] -one of excluding yoga and aquatic exercise- [54] has shown evidence regarding prenatal exercise intervention on increasing frequency of vaginal delivery. Also randomized controlled trials [37,39,58–64] and review on prospective observational studies [65] support our meta-analysis findings.

Regarding to incidence of caesarean and instrumental delivery,maternal and fetal morbidity and mortality are more likely in women who have cesarean sections or instrumental delivery [66] and a higher chance of needing to be readmitted to the hospital. Prenatal exercise may lessen these risks since it lowers the likelihood of cesarean births.

Studies primarily focusing on the effect of physical exercise on the incidence of cesarean delivery have shown that the risk of cesarean section appears to decrease if pregnant women perform regular exercise, which is consistent with our results [8,56,67,68]. However, some studies reported that aerobic exercise intervention has shown no benefit in reducing the incidence of cesarean delivery [69,70].Additionally, although our study revealed that prenatal exercise had no significant effect on the risk of instrumental delivery, other studies have shown evidence that prenatal exercise significantly reduces this risk [8,56].

The advantages of physical activity in other areas for pregnancy were also examined, in addition to its positive effects on delivery outcomes. Exercise has only been shown to shorten the mean duration of first stage of labor (WMD −61.30[−80.63, −41.97) [p = 0.00 at 95% CI]] but has no association with second stage of labor. In line with our findings, several studies found that physical exercise reduces the duration of the first stage of labor [35,71]. While our review found no association between duration of second stage of labor and other primary studies have similar findings [9]. The population, the nature and extent of the intervention, compliance, and dropout rate varied between studies. Even studies with only a few hours of physical activity and a brief intervention period helped to lower the risk of cesarean delivery and improve the frequency of vaginal birth. Therefore, encouraging all pregnant women to engage in physical activity, even in small amounts, is important.

## Strength and limitations

To the best of our knowledge, this is the largest and the most updated review of RCTs conducted on the topic till date. Therefore our results are more precise than the earlier conducted reviews. The variety of databases we employed, along with the wide range of search phrases, led to a very sensitive search since we searched several complimentary electronic databases. Most of the studies included had low to moderate risk of bias (16 RCTs), we detected publication bias for the pooled effect of exercise on Normal Vaginal delivery, thus RR was only reported only after making adjustment by trim and fill analysis.

However, our meta-analysis had several limitations Most of the studies included in the review were of small sample size. The systematic review search did not include unpublished sources or trial registry. Despite employing a thorough search technique, the majority of included studies came from middle and higher income nations; this means that our results may not be applied to low-income countries.

## Conclusion and recommendation

In this meta-analysis, we pooled results from 16 studies with a variety of exercise interventions. Our findings build on existing literature, providing updated and strengthened evidence supporting an increased rate of normal delivery by 14% and a decreased rate of cesarean delivery by 34%. The results contribute to the ongoing discussion on prenatal care by offering a clearer synthesis of RCT data, affirming that physical activity during pregnancy is a beneficial, non-invasive strategy to improve delivery outcomes.

Given the consistent positive associations observed, healthcare providers are encouraged to promote safe, moderate-intensity physical activity as a key component of prenatal care, tailored to each woman’s health status. Future research should aim to explore individualized exercise protocols and assess long-term maternal and neonatal outcomes to further refine guidelines.

## Supporting information

S1 File Searching strategy. (PDF)

S2 File The PRISMA 2020 check list. (DOCX)

S3 File Summary of risk of bias of included studies with supportive judgment of authors’ (n = 16). (PDF)

S4 File Descriptions of 814 excluded records and reasons. (XLSX)

S5 File Descriptions of studies included in the meta-analysis. (XLSX)

## Abbreviations:

ACOG: American Congress of Obstetricians and Gynecologists
Apgar: Appearance, Pulse, Grimace, Activity, and Respiration
GA: Gestational Age
GRADE: Grading of Recommendations Assessment, Development and Evaluation
IVD: Instrumental vaginal delivery
MeSH: Medical Subject Headings
PICO: population, intervention, comparator, outcome
PRISMA: Preferred Reporting Item for Systematic review and Meta-Analysis
PROSPERO: International Prospective Register of Systematic Reviews
RCT: Randomized Controlled Trial
RevMan: Review Manager
RMLE: Restricted maximum likelihood estimation
WHO: World Health Organization
