# Supplementary material for: Effects of physical exercise during pregnancy on delivery outcomes: Systematic review and meta-analysis of randomized controlled trials
Source: PLoS One. 2025 Jul 23;20(7):e0326868. doi: 10.1371/journal.pone.0326868 (PMC12286345; doi:10.1371/journal.pone.0326868)
Supplement: S1 File — (PDF) [file pone.0326868.s001.pdf]

Searching strategy

Step-1: The effect of exercise during pregnancy on mode of delivery.

Step-2: Appropriate data bases (PubMed, HINARI, Cochrane, science direct, SCOPUS, EMBASE,) & search engines (Google scholar)

Step-3: Key words : Exercise, pregnancy ,mode of delivery

Step-4: Identifying alternative and synonym words using MeSH browser and Brainstorming as the following:

**MeSH**	**Entry term**	**Combinations**
**Exercise**	Physical activity<br>yoga<br>Aerobic exercise	**Exercise** OR "Physical activity" OR "Aerobic exercise" OR yoga
**Pregnancy**	Gravid	**pregnancy** OR Gravid
**Mode of delivery**	Delivery Obstetric<br>obstetric deliver*	**Mode of deliver*** OR **"**Delivery Obstetric" OR "obstetric deliver*"
**Final combination**		

Step-5: searching

**Data base/websit e**	**keyword**	**synonym**	**Combination(advanced search)**	**articl es**	**date**
Google scholar	**Exercise, pregnancy,**	"Physical activity", yoga,"Aerobic exercise"	With all of the words –effect of exercise on mode of	147	Augu st

	**mode of delivery**	"Delivery, obstetric","obstetric deliver*" OR "mode of deliver*" Gravid	delivery<br>With exact phrase – Exercise<br>With at least one of the words-		24/2023
PubMed			"Exercise"[Mesh][tiab] OR "physical activity"[tiab] OR "aerobic exercise"[tiab] AND "Delivery, Obstetric"[Mesh][tiab] OR "Obstetric Deliver*"[tiab] OR "mode of deliver*"[tiab] AND "Pregnancy"[Mesh][tiab] OR gravid[tiab]	375	August 24/2023
Science direct			(Exercise OR "aerobic exercise" OR "physical activity" OR yoga) AND (pregnancy OR gravid) AND ("Delivery, obstetric" OR "Deliveries, Obstetric" OR "mode of delivery") ("randomized controlled trial")	147	August 24/2023
Cochrane library			**Exercise OR "physical activity" OR "aerobic exercise" OR "yoga" in Title Abstract Keyword AND "Delivery, Obstetric"OR " Obstetric Deliver*" OR "mode of deliver*"in Title Abstract Keyword AND Pregnancy OR "gravid" in**	41	August 25/2023

			Title Abstract Keyword		
HINARI			(Exercise OR "physical activity" OR "aerobic exercise" OR yoga) AND (“Delivery, Obstetric” OR "Obstetric Deliver*” OR "mode of deliver*") AND (Pregnancy OR gravid)	195	August 25/2023
scopus			(Exercise OR "physical activity" OR "aerobic exercise" OR yoga) AND (“Delivery, Obstetric” OR "Obstetric Deliver*” OR "mode of deliver*") AND (Pregnancy OR gravid)	88	September 20/2023
Embase			(Exercise OR "physical activity" OR "aerobic exercise" OR yoga) AND (“Delivery, Obstetric” OR "Obstetric Deliver*” OR "mode of deliver*") AND (Pregnancy OR gravid)	77	September 20/2023
Psyc INFO			(Exercise OR "physical activity" OR "aerobic exercise" OR yoga) AND (“Delivery, Obstetric” OR "Obstetric Deliver*” OR "mode of deliver*") AND (Pregnancy OR gravid)	80	September 22/2023

Total articles	1150	
