# Supplementary material for: Effects of physical exercise during pregnancy on delivery outcomes: Systematic review and meta-analysis of randomized controlled trials
Source: PLoS One. 2025 Jul 23;20(7):e0326868. doi: 10.1371/journal.pone.0326868 (PMC12286345; doi:10.1371/journal.pone.0326868)
Supplement: S2 File — (DOCX) [file pone.0326868.s002.docx]

| **Section and Topic** | **Item #** | **Checklist item** | **Location where item is reported** |
| --- | --- | --- | --- |
| **TITLE** | | |  |
| Title | 1 | Identify the report as a systematic review. | Page number 1 line 1-2 |
| **ABSTRACT** | | |  |
| Abstract | 2 | See the PRISMA 2020 for Abstracts checklist. | Page number 2 line 23-55 |
| **INTRODUCTION** | | |  |
| Rationale | 3 | Describe the rationale for the review in the context of existing knowledge. | Page number 3 and 4 |
| Objectives | 4 | Provide an explicit statement of the objective(s) or question(s) the review addresses. | Page number 3 line 106-108 |
| **METHODS** | | |  |
| Eligibility criteria | 5 | Specify the inclusion and exclusion criteria for the review and how studies were grouped for the syntheses. | Page number 5 line 116-130 |
| Information sources | 6 | Specify all databases, registers, websites, organisations, reference lists and other sources searched or consulted to identify studies. Specify the date when each source was last searched or consulted. | Page number 5 and 6 line 132-144 |
| Search strategy | 7 | Present the full search strategies for all databases, registers and websites, including any filters and limits used. | Page number 5 and 6 line 132-144 |
| Selection process | 8 | Specify the methods used to decide whether a study met the inclusion criteria of the review, including how many reviewers screened each record and each report retrieved, whether they worked independently, and if applicable, details of automation tools used in the process. | Page number 6 line 142-144 |
| Data collection process | 9 | Specify the methods used to collect data from reports, including how many reviewers collected data from each report, whether they worked independently, any processes for obtaining or confirming data from study investigators, and if applicable, details of automation tools used in the process. | Page number 6 line 145-155 |
| Data items | 10a | List and define all outcomes for which data were sought. Specify whether all results that were compatible with each outcome domain in each study were sought (e.g. for all measures, time points, analyses), and if not, the methods used to decide which results to collect. | Page number 6 line 156-162 |
|  | 10b | List and define all other variables for which data were sought (e.g. participant and intervention characteristics, funding sources). Describe any assumptions made about any missing or unclear information. |  |
| Study risk of bias assessment | 11 | Specify the methods used to assess risk of bias in the included studies, including details of the tool(s) used, how many reviewers assessed each study and whether they worked independently, and if applicable, details of automation tools used in the process. | Page number 6 and 7 line 163-173 |
| Effect measures | 12 | Specify for each outcome the effect measure(s) (e.g. risk ratio, mean difference) used in the synthesis or presentation of results. | Page number 6 line 160-162 |
| Synthesis methods | 13a | Describe the processes used to decide which studies were eligible for each synthesis (e.g. tabulating the study intervention characteristics and comparing against the planned groups for each synthesis (item #5)). | Page number 7 line 175-195 |
|  | 13b | Describe any methods required to prepare the data for presentation or synthesis, such as handling of missing summary statistics, or data conversions. | Page number 7 line 175-195 |
|  | 13c | Describe any methods used to tabulate or visually display results of individual studies and syntheses. | Page number 7 line 175-195 |
|  | 13d | Describe any methods used to synthesize results and provide a rationale for the choice(s). If meta-analysis was performed, describe the model(s), method(s) to identify the presence and extent of statistical heterogeneity, and software package(s) used. | Page number 7 line 175-195 |
|  | 13e | Describe any methods used to explore possible causes of heterogeneity among study results (e.g. subgroup analysis, meta-regression). | Page number 7 line 175-195 |
|  | 13f | Describe any sensitivity analyses conducted to assess robustness of the synthesized results. | Page number 7 line 187-189 |
| Reporting bias assessment | 14 | Describe any methods used to assess risk of bias due to missing results in a synthesis (arising from reporting biases). | Page number 7 line 190-195 |
| Certainty assessment | 15 | Describe any methods used to assess certainty (or confidence) in the body of evidence for an outcome. | Page number 8 line 197-203 |
| **RESULTS** | | |  |
| Study selection | 16a | Describe the results of the search and selection process, from the number of records identified in the search to the number of studies included in the review, ideally using a flow diagram. | Page number 9 line 219-227 |
|  | 16b | Cite studies that might appear to meet the inclusion criteria, but which were excluded, and explain why they were excluded. | Page number 9 line 219-227 |
| Study characteristics | 17 | Cite each included study and present its characteristics. | Page number 9 and 10 line 231-275 |
| Risk of bias in studies | 18 | Present assessments of risk of bias for each included study. | Page number 11 line 277-284 |
| Results of individual studies | 19 | For all outcomes, present, for each study: (a) summary statistics for each group (where appropriate) and (b) an effect estimate and its precision (e.g. confidence/credible interval), ideally using structured tables or plots. | Page number 11 -13 line 290-344 |
| Results of syntheses | 20a | For each synthesis, briefly summarise the characteristics and risk of bias among contributing studies. | Page number 11 -13 line 290-344 |
|  | 20b | Present results of all statistical syntheses conducted. If meta-analysis was done, present for each the summary estimate and its precision (e.g. confidence/credible interval) and measures of statistical heterogeneity. If comparing groups, describe the direction of the effect. | Page number 11 -13 line 290-344 |
|  | 20c | Present results of all investigations of possible causes of heterogeneity among study results. | Page number 11 -13 line 290-344 |
|  | 20d | Present results of all sensitivity analyses conducted to assess the robustness of the synthesized results. | Page number 11 -13 line 346-349 |
| Reporting biases | 21 | Present assessments of risk of bias due to missing results (arising from reporting biases) for each synthesis assessed. | Page number 13 line 353-361 |
| Certainty of evidence | 22 | Present assessments of certainty (or confidence) in the body of evidence for each outcome assessed. | Page number 13-14 line 366-368 |
| **DISCUSSION** | | |  |
| Discussion | 23a | Provide a general interpretation of the results in the context of other evidence. | Page number 15 and16 line 381-433 |
|  | 23b | Discuss any limitations of the evidence included in the review. | Page number 15 and16 line 381-433 |
|  | 23c | Discuss any limitations of the review processes used. | Page number 15 and16 line 381-433 |
|  | 23d | Discuss implications of the results for practice, policy, and future research. | Page number 15 and16 line 381-433 |
| **OTHER INFORMATION** | | |  |
| Registration and protocol | 24a | Provide registration information for the review, including register name and registration number, or state that the review was not registered. | Page number 5 line 112 |
|  | 24b | Indicate where the review protocol can be accessed, or state that a protocol was not prepared. | Page number 5 line 111 |
|  | 24c | Describe and explain any amendments to information provided at registration or in the protocol. |  |
| Support | 25 | Describe sources of financial or non-financial support for the review, and the role of the funders or sponsors in the review. | Page number 19 line 493 |
| Competing interests | 26 | Declare any competing interests of review authors. | Page number 19 line 495 |
| Availability of data, code and other materials | 27 | Report which of the following are publicly available and where they can be found: template data collection forms; data extracted from included studies; data used for all analyses; analytic code; any other materials used in the review. | Page number 19 line 490-491 |

*From:*  Page MJ, McKenzie JE, Bossuyt PM, Boutron I, Hoffmann TC, Mulrow CD, et al. The PRISMA 2020 statement: an updated guideline for reporting systematic reviews. BMJ 2021;372:n71. doi: 10.1136/bmj.n71
