# Supplementary material for: Effects of physical exercise during pregnancy on delivery outcomes: Systematic review and meta-analysis of randomized controlled trials
Source: PLoS One. 2025 Jul 23;20(7):e0326868. doi: 10.1371/journal.pone.0326868 (PMC12286345; doi:10.1371/journal.pone.0326868)
Supplement: S3 File — (PDF) [file pone.0326868.s003.pdf]

Table 3.summary of risk of bias of included studies with supportive judgment of authors' (n=16).

1. **Barakat et al, 2011**

**Bias domain**	**Source of bias**	**Author's Judgment**	**Support for judgment**
Selection bias	Sequence generation	**Unclear**	Not enough information to categorize
	Allocation concealment	**Low risk**	Randomization was concealed using random number table
**Performance bias**	Binding of participants and personnel*	**Low risk**	Eventhough method of blinding was not mentioned The nature of the study does not permit blinding of participant and outcome couldn't be likely to be influenced
**Detection bias**	Blinding of outcome assessment	**Unclear**	No clear description about masking
**Attrition bias**	Incomplete outcome data	**Low risk**	Few dropout comparably on both group with no relation to outcome of interest
**Reporting bias**	Selective reporting	**Low risk**	The study protocol is not available but it is clear that the published reports include all expected outcomes, including those that were pre-specified in the method
**Other bias**	Anything else, ideally pre-specified	**Low risk**	Expected variables that are related to outcome were included at baseline with no significance difference among groups
**Score**	5 Low risk + 2 Unclear == fair quality		

2. **Barakat et al,2012(A)**

**Bias domain**	**Source of bias**	**Author's Judgment**	**Support for judgment**

Selection bias	Sequence generation	**Low risk**	computer-generated list of random numbers was used
	Allocation concealment	**unclear risk**	Sequence concealed but do not explain how
**Performance bias**	Binding of participants and personnel*	**Low risk**	Blinding of participants was not stated but less likely to be influenced
**Detection bias**	Blinding of outcome assessment	**Low risk**	outcome assessors were blinded
**Attrition bias**	Incomplete outcome data	**Low risk**	no lost to follow up between groups
**Reporting bias**	Selective reporting	**Low risk**	The study protocol is not available but it is clear that the published reports include all expected outcomes, including those that were pre-specified in the method
**Other bias**	Anything else, ideally pre-specified	**Low risk**	no baseline imbalance between groups, Confounding well controlled at baseline and analysis
**Score**	6 Low risk + 1 Unclear == Good quality		

3. **Barakat et al, 2012 (B)**

Objective: The aim of the present study was to assess the influence of a physical activity program with land/aquatic activities during pregnancy on the 50 g MGS at 24–28 week

**Bias domain**	**Source of bias**	**Author's Judgment**	**Support for judgment**

Selection bias	Sequence generation	**Unclear**	Sequence generation is not described
	Allocation concealment	**High risk**	The method of randomization is not clearly described
**Performance bias**	Binding of participants and personnel*	**Unclear**	Even though method of masking participant was not mentioned The nature of the study does not permit blinding of participant and outcome couldn't be likely to be influenced
**Detection bias**	Blinding of outcome assessment	**Unclear**	No description about masking of outcome assessor
**Attrition bias**	Incomplete outcome data	**Low risk**	Few dropout comparably on both group with no relation to outcome of interest
**Reporting bias**	Selective reporting	**Low risk**	The study protocol is not available but it is clear that the published reports include all expected outcomes, including those that were pre-specified in the method
**Other bias**	Anything else, ideally pre-specified	**Low risk**	Expected variables that are related to outcome were included at baseline with no significance difference among groups
**Score**	3 Low risk + 3 Unclear + 1 High risk== Poor quality		

4. **Barakat et al. 2013**

Objective: The aim of the present study was to examine the influence of a program of moderate physical exercise throughout pregnancy on maternal and fetal parameters

Primary outcome: Maternal and fetal Outcomes

**Bias domain**	**Source of bias**	**Author's Judgment**	**Support for judgment**
Selection bias	Sequence generation	**Low risk**	For allocation of the participants, a computer-generated list of random numbers was used
	Allocation	**High risk**	Method of Allocation concealment is not

	concealment		described
**Performance bias**	Binding of participants and personnel*	**Low risk**	The nature of the study does not permit blinding of participant and outcome unlikely to be influenced
**Detection bias**	Blinding of outcome assessment	**Unclear**	No clear description about masking
**Attrition bias**	Incomplete outcome data	**Low risk**	Comparable among groups and ITT was used for analysis
**Reporting bias**	Selective reporting	**Low risk**	The study protocol is not available but it is clear that the published reports include all expected outcomes, including those that were pre-specified in the method
**Other bias**	Anything else, ideally pre-specified	**Low risk**	Confounding well controlled at baseline and analysis
**Score**	4 Low risk + 1 High risk + 2 Unclear == Fair quality		

5. **Barakat et al. 2016**

**Bias domain**	**Source of bias**	**Author's Judgment**	**Support for judgment**
**Selection bias**	Sequence generation	**unclear**	Sequence generation not described
	Allocation concealment	**Low risk**	Sequence was generated using random number table
**Performance bias**	Blinding of participants and personnel	**Low risk**	The nature of the study does not permit blinding of participant and outcome couldn't be likely to be affected
**Detection bias**	Blinding of outcome assessment	**Low risk**	Outcome assessor were blinded
**Attrition bias**	Incomplete outcome	**Low risk**	Comparable and very few loss to follow up

	data		which is below 9 %
**Reporting bias**	Selective reporting	**Low risk**	The study protocol is not available but it is clear that the published reports include all expected outcomes, including those that were pre-specified in the method
**Other bias**	Anything else, ideally pre-specified	**High risk**	Important confounding variables related with outcome were not included at baseline
**Score**	5 low risk + 1 unclear + High risk == Fair quality		

6. **Barakat et al. 2018**

**Bias domain**	**Source of bias**	**Author's Judgment**	**Support for judgment**
Selection bias	Sequence generation	low risk	Computer generated list of random numbers was used to allocate the participants into the study groups
	Allocation concealment	**unclear risk**	Sequence concealed ,but the method of concealment not explained
**Performance bias**	Binding of participants and personnel*	**Low risk**	participants were blinded
**Detection bias**	Blinding of outcome assessment	**Low risk**	outcome assessors were also blinded
**Attrition bias**	Incomplete outcome	**Low risk**	Intent to treat Analysis was used, no significant

	data		imbalance between groups.
**Reporting bias**	Selective reporting	**Low risk**	primary and secondary outcome clearly stated according to prespecified protocol
**Other bias**	Anything else, ideally pre-specified	**Low risk**	no baseline imbalance<br>Expected variables that are related to outcome were included at baseline
**Score**	6 Low risk + 1 Unclear == Good quality		

7. centha et al. 2018

**Bias domain**	**Source of bias**	**Author's Judgment**	**Support for judgment**
Selection bias	Sequence generation	**Low risk**	They were randomized by using the minimized randomization program
	Allocation concealment	**unclear risk**	it is not stated weather is was concealed or not
**Performance bias**	Binding of participants and personnel*	**Low risk**	single blinding was ,participant were were blinded
**Detection bias**	Blinding of outcome assessment	**unclear risk**	outcome assessors blinding were not explained
**Attrition bias**	Incomplete outcome data	**Low risk**	lost to follow up is balanced between groups and less than 20%
**Reporting bias**	Selective reporting	**Low risk**	The study protocol is not available but it is clear that the published reports include all expected outcomes, including those that were pre-specified in the method
**Other bias**	Anything else,	**Low risk**	no baseline imbalance between groups

	ideally pre-specified		
**Score**	5 Low risk + 2 Unclear == fair quality		

8. carascossa et al. 2018

**Bias domain**	**Source of bias**	**Author's Judgment**	**Support for judgment**
Selection bias	Sequence generation	**Low risk**	A computer-generated randomization list in blocks of 6 was used to randomly allocate women to each group. The details of the 1:1 randomization and centralized allocation process
	Allocation concealment	**unclear risk**	sequence concealed ,but it is not explained how it was done
**Performance bias**	Binding of participants and personnel*	**Low risk**	Not stated but less likely to be affected by blinding.
**Detection bias**	Blinding of outcome assessment	**Low risk**	Evaluators and external personnel were blinded
**Attrition bias**	Incomplete outcome data	**Low risk**	lost to follow up is not imbalanced between groups
**Reporting bias**	Selective reporting	**unclear risk**	not enough information to categorize
**Other bias**	Anything else, ideally pre-specified	**Low risk**	no baseline imbalance between groups
**Score**	5 Low risk + 2 Unclear == fair quality		

9. Hakastad et al. 2020

**Bias domain**	**Source of bias**	**Author's Judgment**	**Support for judgment**
Selection bias	Sequence generation	**Low risk**	A simple computer-based randomization program
	Allocation concealment	**Low risk**	Allocations were sealed in opaque numbered envelopes
**Performance bias**	Binding of participants and personnel*	**Low risk**	Not stated but less likely to be affected by blinding.
**Detection bias**	Blinding of outcome assessment	**Low risk**	primary investigator were blinded
**Attrition bias**	Incomplete outcome data	**Low risk**	lost to follow up was<20% and ITT analysis was used
**Reporting bias**	Selective reporting	**Low risk**	Pre specified outcomes have been stated according to the consort protocol statements
**Other bias**	Anything else, ideally pre-specified	**Low risk**	no baseline difference difference beteewn
**Score**	7 Low risk == Good quality		

10 . Jahdi et al. 2016

**Bias domain**	**Source of bias**	**Author's Judgment**	**Support for judgment**
Selection bias	Sequence generation	**Low risk**	A simple-random approach, a table of random numbers was used.

	Allocation concealment	**Low risk**	Allocations were sealed in opaque numbered envelopes
**Performance bias**	Binding of participants and personnel*	**Low risk**	Not stated but less likely to be affected by blinding.
**Detection bias**	Blinding of outcome assessment	**Low risk**	primary investigator were blinded
**Attrition bias**	Incomplete outcome data	**Low risk**	lost to follow up was<20% and ITT analysis was used
**Reporting bias**	Selective reporting	**Unclear risk**	insufficient information to categorize
**Other bias**	Anything else, ideally pre-specified	**Unclear risk**	Analysis not explained
**Score**	5 Low risk+2 unclear == fair quality		

11. **mohaydin et al. 2020**

**Bias domain**	**Source of bias**	**Author's Judgment**	**Support for judgment**
Selection bias	Sequence generation	**high risk**	Sequence generated by odd or even date
	Allocation concealment	**unclear risk**	Allocations method was not explained
**Performance bias**	Binding of participants and personnel*	**Low risk**	Not stated but less likely to be affected by blinding.

**Detection bias**	Blinding of outcome assessment	**unclear risk**	Blinding not explained
**Attrition bias**	Incomplete outcome data	**High risk**	loss of follow up not imbalanced between groups
**Reporting bias**	Selective reporting	**Unclear risk**	insufficient information to categorize
**Other bias**	Anything else, ideally pre-specified	**Unclear risk**	Analysis not explained
**Score**	1 Low risk+4 unclear+ 2 high risk == poor quality		

12. **Price et al. 2012**

**Bias domain**	**Source of bias**	**Author's Judgment**	**Support for judgment**
Selection bias	Sequence generation	**low risk**	Randomized using table of random numbers
	Allocation concealment	**Low risk**	opaque envelopes containing an equal number of group assignment
**Performance bias**	Binding of participants and personnel*	**Low risk**	Not stated but less likely to be affected by blinding.
**Detection bias**	Blinding of outcome assessment	**unclear risk**	primary investigator blinding not explained
**Attrition bias**	Incomplete outcome data	**Low risk**	loss of follow up not imbalanced between groups
**Reporting bias**	Selective reporting	**Unclear risk**	insufficient information to categorize
**Other bias**	Anything else, ideally pre-specified	**low risk**	No imbalance between groups

**Score**	5 Low risk+2 unclear == fair quality

13. **Yekefallah et al. 2021**

**Bias domain**	**Source of bias**	**Author's Judgment**	**Support for judgment**
Selection bias	Sequence generation	**High risk**	Sequence generation were not used in randomization
	Allocation concealment	**High risk**	Participants were assigned to either groups using non-random method (applying Odd days to assign control and even days to Intervention)
**Performance bias**	Binding of participants and personnel*	**Low risk**	The nature of the study does not permit blinding of participant and outcome unlikely to be influenced
**Detection bias**	Blinding of outcome assessment*	**Unclear**	Masking of assessor were not specified
**Attrition bias**	Incomplete outcome data*	**Low risk**	No missing data
**Reporting bias**	Selective reporting	**Unclear**	Protocol is not available
**Other bias**	Anything else, ideally pre-specified	**High risk**	
**Score**	2 low risk + 3 high risk + 2 Unclear== poor quality		

14. **Ghandali et al. 2016**

**Bias domain**	**Source of bias**	**Author's Judgment**	**Support for judgment**

Selection bias	Sequence generation	**Low risk**	Random sequence generation with block of 6 was performed
	Allocation concealment	**Low risk**	Participants were Randomly assigned using closed envelops
**Performance bias**	Binding of participants and personnel*	**Low risk**	The nature of the study does not permit blinding of participant and outcome couldn't be likely to be affected
**Detection bias**	Blinding of outcome assessment*	**Low risk**	Outcome evaluator was not involved in randomization and blinded to group assignment
**Attrition bias**	Incomplete outcome data*	**Low risk**	Comparable(i.e. 4 & 3 dropout among intervention and control group) and not associated with outcome
**Reporting bias**	Selective reporting	**Low risk**	Eventhough Protocol is not feasible all pre-specified outcome in the method were reported
**Other bias**	Anything else, ideally pre-specified	**Low risk**	Confounding well control at randomization and analysis
**Score**	4 low risk = Good quality		

15. **Sliviera et al. 2012**

**Bias domain**	**Source of bias**	**Author's Judgment**	**Support for judgment**
**Selection bias**	Sequence generation	**High risk**	Sequence was not generated
	Allocation concealment	**High risk**	Non-random Subjects selection (putted as limitation)
**Performance bias**	Binding of participants and personnel	**Low risk**	Random sequence generation with block of 30 was performed

**Detection bias**	Blinding of outcome assessment	**Low risk**	The research assistance was not involved and blinded during randomization
**Attrition bias**	Incomplete outcome data	**Low risk**	Comparable and very low (i.e. 8 & 9 dropout respectively in Intervention and control group) and the analyses were conducted using a modified intention to treat analysis
**Reporting bias**	Selective reporting	**Low risk**	The study protocol is not available but it is clear that the published reports include all expected outcomes, including those that were pre-specified in the method
**Other bias**	Anything else, ideally pre-specified	**High risk**	Baseline characteristics of participant is not mentioned indicating chance of difference among groups
**Score**	2 unclear + 5 low risk == fair quality		

16. Perales et al. 2014

**Bias domain**	**Source of bias**	**Author's Judgment**	**Support for judgment**
Selection bias	Sequence generation	**Low risk**	For allocation of the participants, a computer-generated list of random numbers was used
	Allocation concealment	**High risk**	Method of Allocation concealment is not described
**Performance bias**	Binding of participants and personnel*	**Low risk**	The nature of the study does not permit blinding of participant and outcome unlikely to be influenced
**Detection bias**	Blinding of outcome assessment	**Unclear**	No clear description about masking of Assessor
**Attrition bias**	Incomplete outcome data	**Low risk**	Comparable among groups and ITT was used for analysis
**Reporting bias**	Selective reporting	**Low risk**	The study protocol is not available but it is clear

			that the published reports include all expected outcomes, including those that were pre-specified in the method
**Other bias**	Anything else, ideally pre-specified	**Low risk**	Confounding well controlled at baseline and analysis
**Score**	4 Low risk + 1 High risk + 2 Unclear == Fair quality		
